# The Prestige Elite in Sociology: Toward a Collective Biography of the Most Cited Scholars (1970-2010)

**DOI:** 10.1080/00380253.2019.1581037

**Published:** 2019-04-16

**Authors:** Philipp Korom

**Affiliations:** Department of Sociology, University of Graz, Graz, Austria

**Keywords:** Prestige elite, sociology, eminence, bibliometrics, collective biography

## Abstract

This study is the first to systematically identify the most recognized scholars in sociology in the 1970s and 2010s by citation counts. This is achieved on the basis of a newly generated text corpus of approximately 49,000 pages, which encompasses various genres of literature (encyclopedias, handbooks, journals, textbooks). Investigations into common characteristics reveal that, in the 1970s, elites typically received their PhD from Columbia University, Harvard University, or the University of Chicago. The contemporary elite is partly European. In general, eminence is short-lived (<40 years). Over time, the elite has remained socially heterogeneous, but becomes more mobile and increasingly moves between universities. Coverage in specialist and generalist journals suggests that elite status in sociology cannot be achieved simply by dominating multiple communities inside sociology; elite sociologists are typically well received in the discipline’s core.

## Introduction

There is abundant evidence that stratification exists within very different scientific disciplines (Cole and Cole 1973). Once in the academy, every scholar is thrown into an economy grounded in the collection of prestige: awards, grants, publications, invitations to talk, and other items that add lines to a curriculum vitae (English 2005). Consequently, scholars are judged by other members of the academy in terms of the quality and quantity of prestige items accrued. While different criteria in the judgement of academic excellence exist (Tsay et al. 2003), it is evident that a scholar’s overall academic prestige or reputation will determine his professional status.

Several studies have found steep academic hierarchies within sociology with regard to interdepartmental prestige hierarchies, citation counts, editorships in “top-tier journals,” and areas of specialization. The prestige gap between elite and nonelite sociology departments in U.S. universities has been proven to be enduring (Weakliem, Gauchat, and Wright 2012). The top five sociology departments hire about 90% of their students from the top 20 schools (Burris 2004). Across very different scientific fields, including sociology, highly cited work proves to be more strongly based on previously highly cited papers than on medium-cited work (Bornmann, Anegón, and Leydesdorff 2010). An elite stratum dominates the editorial boards of top journals (Yoels 1971), and some subfields in sociology enjoy much broader reputation than others (Lee, Runda, and Lee 1974), which further hints at an intradisciplinary status hierarchy (Weeber 2006).

What remains under-researched, however, is the discipline’s prestige elite, that is the “typically thin layer of people […] who generally have the highest prestige within what is prestigious collectivity to begin with” (Zuckerman 1972:159). In contrast to other disciplines such as economics (Bjork, Offer, and Söderberg 2014), political science (Bingham and Vertz 1983) or psychology (Diener, Oishi, and Park 2014), the sociological literature does not speak to the issue of disciplinary elites. The sparse work on eminence in sociology is confined to case studies on single scholars (e.g., Sallaz and Zavisca 2007).

One can only speculate on the reasons for this blatant research gap, but crucial factors could be the perceived fractious character of the discipline DiMaggio (1997:189) as well as the pronounced structuring by language and national frames. Given the “pluralistic mosaic of sociology” (Sztompka 2010:24) with its many areas of specialization (e.g., criminology, social stratification research, etc.) and national traditions, a consensus on the question of who belongs to the—most likely heterogeneous—elite in an internally balkanized discipline appears to many impossible to reach.

This study rejects the notion that it is impossible to determine elite status in sociology and begins from the assumption that citations are the best available measure of academic prestige if a variety of literature genres is considered. Based on citation analysis of a newly generated, vast text corpus, which includes encyclopedias, handbooks, journals, and textbooks, about 50 scholars have been identified for both the 1970s and 2010s as members of the prestige elite.^1^ Several robustness checks are performed to ascertain the validity of the selection process and information on honorific society membership and prestigious awards is used to corroborate the accuracy of the “citation approach.”

Further first steps in the direction toward a “collective biography” (Charle 2015) of the academic elite in sociology are made by analyzing the family background, academic careers, and reception patterns in the diverse world of academic journals.

## Prestige Elites in Sociology

In academia, as in other societal domains, the uppermost layer consists of a numerical minority that is often referred to as the “elite.” The concept of elite suffers from semantic confusion (Hartmann 2007). One research tradition goes back to Vilfredo Pareto, who was the first to use the term for small groups of people standing out from the overall population due to their superior achievement.^2^

With regard to science, there is evidence that “the perceived ‘quality’ of a scientist’s work remains in general the most significant determinant of recognition” (Cole 1992:171) and that recognition by peers is key in acquiring elite status (Zuckerman 1977). Particularistic elements (e.g., having powerful mentors) have a role, albeit a limited one (Cao 2004; Lutter and Martin 2016). The core characteristic of prestige elites in science is thus that they are honored by knowledgeable peers. The professional community honors the few members of the elite for their academic achievements in various ways, from eponymy and prestigious awards, to membership in honorific societies, fellowships, and honorary degrees, to citations (Mulkay 1976). The extent of professional reputation is determined, among other things, by two central factors (Speier 1935): the number of persons familiar with the claim that honor is to be done and, more directly, the number of those who are willing to pay it. Members of elites are thus those who achieve the height of inconspicuous eminence because most peers clearly recognize their outstanding contributions to the advancement of scientific knowledge.

Given these insights, scholars in the uppermost ranks can be referred to as prestige elites. To some extent, prestige elites might as well be power elites that have a (political) impact on the organization of science and research (“prestige-power elites”). In many cases, however, prestige elites are distinct from power elites in academia.^3^ Moreover, prestige elites in the sense used here are conceptually unconnected to elite departments or competitive research funding; the key determinant of elite status is the reception of scholars’ work by peer audiences.

## How to Identify Prestige Elites?

Differences in the contribution to the advancement of knowledge are difficult to judge as there exist divergent theoretical views on what criteria should be applied to identify leading achievements (Deutsch, Markovits, and Platt 1986; Rule 1997). In early eminence research, peer review—in which scientific quality is judged by other scientists (“peers”)—was the prime method for determining scientific quality. For example, James McKeen Cattell, the editor of *Science*, sought to identify leading scientists by asking 10 eminent scientists to rank their contemporaries in order of merit.^4^ Today, the quality of the most prestigious grants is decided by peer review and eminent peers decide to whom the Nobel Prize, or other outstanding awards, should be given.

Peer-review processes mostly retain the characteristics of a “black box” (Sonnert 1995). More importantly, peers do not agree reliably on scientific quality (Bornmann, Mutz, and Daniel 2010). Empirical studies on peer review panels reveal that peer “evaluation is not based on stable comparables, and that various competing criteria with multiple meanings are used to assess academic work” (Lamont 2009:18).

In general, peer judgements are not easily available. Thus instead of asking experts to derive lists of the most recognized peers,^5^ eminence researchers often rely on prestigious awards as an indicator of the outstanding quality of a researcher (Diener, Oishi, and Park 2014). In the case of sociology, there are a considerable number of international awards, such as European Amalfi Prize, the Holberg Prize, or the Princess of Asturias Award. While the selection is always made by a jury that includes distinguished social scientists, the nomination processes, prize amounts, award categories (e.g., life work, past research) and boundaries of eligibility vary hugely. Given the fact that no prize has yet reached the high reputation and public awareness of the Nobel Prize (in economics), or the Canadian Fields Medals (in mathematics), and that most sociological awards were established relatively recently, these manifestations of scholarly recognition do not appear to be the best indicator of elite status in sociology.

Another high honor that a scientist can receive is membership of an Academy (Cao 2004). Academies might function simply as honorific societies (e.g., Royal Society of London; National Academy of Sciences), or they might combine research with honorary activities (e.g., French Academy of Sciences). In general, only Academy members can submit formal nominations and candidates are admitted only after having passed a vetting process. However, given the high number of social science fellows and foreign or honorary members in the Academies around the world, being elected into one or many Academies may only serve as a rough “proxy” for belonging to the academic elite.

The only remaining indicator for outstanding scientific quality are citation counts. While a general consensus exists that informed peer review cannot be fully replaced by citations analysis (Warner 2000), the evidence that citations correlate highly with various types of scientific recognition is strong and consistent (Cole and Cole 1973). Further, while it is the case that research is cited for reasons other than quality, this argument becomes difficult to maintain when leveled at citation elites (Parker, Lortie, and Allesina 2010).

The most powerful evidence for an association between scientific quality and citation counts was delivered by Eugene Garfield, who launched the (Social) Science Citation Index (SSCI). He established that at his time of writing of the most-cited 50 economists 17 had won the Nobel prize (Garfield 1990). Another eight listed scholars received the prize after the publication of Garfield’s study. Thus the “hit rate” has reached 50%. Overall, his results on Nobel Prize winners allow him to conclude that “a simple, quantitative, and objective algorithm based on citation data can effectively corroborate—and even forecast—a complex, qualitative, and subjective selection process based on human judgement” (Garfield and Welljams-Dorof 1992:117).^6^

Citations in journals and textbooks form the foundation of the few existing studies on eminence in sociology (Bain 1962; Oromaner 1980). To date, only one single study established eminence in sociology based on citation rankings derived equally from monographic and journal literature (Cronin, Snyder, and Atkins 1997). Following Cronin et al., the present study takes into account that there is a variety of literature genres in sociology and attempts to capture elite status by analyzing citations in books, encyclopedias, journals, and textbooks.

## Four Sets of Research Questions

Despite the exploratory character of this first study on the prestige elite in sociology, research is guided by classical research questions posed in elite research as well as by preliminary ideas about the changing character of elites between 1970 and 2010.

1. *Sociobiographical profile: Does academic elite status depend on family background?*

The first set of research questions focuses on the family background of elites. It is one of the core assumptions in social stratification research that parents’ socioeconomic status determines, at least to some degree, the professional careers of their children. Knowledge on the social origin of academic elites is, however, extremely scarce. There is some limited evidence that suggests Nobel Laureates in the sciences remain concentrated in families that can provide their offspring with a “head start in access to system-recognized opportunities” (Zuckerman 1977:68). I thus assume that academic elites come from higher social strata.

2. *Institutional affiliations: Which institutions make academic elites in sociology?*

The institutional context greatly influences how academics set their preferences, including recognition sought, perceptions of reward structures, and research performance (Hermanowicz 2009). Elite institutions that value research above all else and hire only highly adept faculty are very likely to instill the will to “strive for the stars” in science. Other advantages of top-tier departments are the ease of access to relevant data or literature, a higher likelihood of being granted research grants (Hönig 2017), the ease of building social ties to important gatekeepers or simply the balance of time allocated to research and teaching within the department. Give these advantages, much speaks in favor of the thesis that few elite institutions host most eminent scholars.

3. *Nation boundedness: Has the academic elite become more European and less American?*

In some sense, academic elites are global. Elite scholars write articles and books that everyone reads and talks about and they generally attract attention throughout the globe. The geographical distribution of academic elites is, however, very uneven (Parker, Lortie, and Allesina 2010). In the case of sociology, much suggests that in the past elite sociologists were principally situated in the United States. The world’s first department of sociology was founded at the University of Chicago in 1892 with millions of dollars of support from the Rockefeller foundation and the so-called Chicago School of Sociology established a professional dominance in the discipline (Cortese 1995). After the Second World War, the universities Columbia, Harvard and Berkeley started to rival Chicago in terms of faculty quality (Weakliem et al. 2012) and a certain Americanization of European social theory was observed. Today, however, the works that catch most attention are contributed by European theorists (e.g., P. Bourdieu, A. Giddens), which suggests “another Golden Era of European Sociology” (Nedelmann and Sztompka 1993:1). I thus hypothesize that the breeding ground of academic elites in sociology has shifted, at least partly, from the United States to Europe.

4. *Reception in the literature: How visible is the work of elite sociologists across nations and the various branches of sociology?*

In contrast to the average academic’s publications, the work of eminent sociologists is received in very different regional settings and disciplinary frameworks. It is, for example, well documented that Bourdieu’s theoretical concepts have circulated in such different research fields as cultural consumption or sociology of education and have become as influential in the United States as in Continental Europe (Santoro, Gallelli, and Barbara 2018). It is thus reasonable to assume that the work of academic elites is not confined to single nations or subfields of sociology.

## Text Corpus, Register of Eminent Sociologists, and Research Strategy

### Text Corpus

This study is based on a text corpus that covers two encyclopedias, two handbooks, five “top” journals and 10 textbooks from either the 1970s or 2010s (see Appendix A). Analyzing literature with publication dates 40 years apart allows to examine the compositional change in sociology’s prestige elite. Many of the 1970s authors were no longer active by 2010, and the leading theories of the 1970s (e.g., functionalism) had lost considerable currency by 2010. Moreover, previous investigations into the most cited sociologists clearly reveal changing orders of prestige over the time analyzed here (Halsey 2004).^7^

The analyzed text belongs to one of the following four genres of literature (see Table 1).

1. *Journals*: The present sample includes two “major” journals (*American Sociological Review* [ASR] and *American Journal of Sociology* [AJS]) of American sociology and one semi-major journal (*Social Forces* [SF]). I further considered the *British Journal of Sociology* (BJS) and the *European Journal of Sociology* (EJS); two European generalist journals that were already firmly established in the 1970s. For each journal, I considered every article published in all volumes of the years 1970 and 2010; a total of 995 articles.^8^
2. *Textbooks*: As it is impossible to select the most widely used textbooks based on sales figures due to a lack of information, I had to settle on an alternative proxy for sales. The digital tool WorldCat indicates the number of libraries in which a certain textbook is available and this became the present main selection criterion.
3. *Handbooks*: Handbook chapters (e.g., “Political Sociology” or “Theory of Organizations”) give an overview of the various subfields of sociology and include representative literature for each of these subfields and reference many monographs.
4. *Encyclopedias*: As encyclopedias of sociology such as *the Blackwell Encyclopedia of Sociology* became available only recently, I decided to include two editions of the *International Encyclopedia of the Social (and Behavioral) Sciences*, which covers all social sciences. The encyclopedic material alone constitutes a large text corpus. Sills (1968) contains 1,716 articles by 1,505 contributors and Wright (2015) features approximately 4,000 entries by 4,945 contributors.

**Table 1.** Composition of the text corpus (page count for each literature genre).

	**1970**	**2010**
*Journals*	17.8%	16.7%
AJS	912	1,512
ASR	687	944
SF	526	1,950
BJS	389	675
EJS	371	422
*Textbooks*	15.2%	10.9%
Bierstedt(1974)	579	
Broom and Selznick (1973)	653	
Horton and Hunt (1964)	581	
Inkeles (1964)	120	
Lenski (1970)	525	
Giddens (2009)		1,194
Henslin (2014)		502
Kendall (2012)		741
Macionis (2012)		670
Schaefer (2012)		470
*Handbooks*	6.7%	1.8%
Faris (1964)	1,088	
Calhoun, Rojek and Turner (2005)		590
*Encyclopedias*	60.3%	70.6%
Sills (1968)	9,750	
Wright (2015)		23,185
*Total*	16,181	32,855

*Note*. Percentage numbers indicate the source-specific share of pages. AJS = *American Journal of Sociology*; ASR = *American Sociological Review*; SF = *Social Forces*; BJS = *British Journal of Sociology*; EJS = *European Journal of Sociology*.

As indicated in Table 1, the text corpus contains 49,036 pages and is not balanced; encyclopedias contribute the most pages and handbooks the least. Rather than aiming at a balanced representation of different text types, the rationale behind the creation of the text corpus was to include text sources in which citations stand for different types of peer recognition.

Citations in textbooks and encyclopedias are likely to indicate that someone is judged by peers to have contributed “certified knowledge” (Merton 1974) to the core of the discipline, which has been commonly approved by at least one generation of scholars.

As handbooks are authoritative guides to different subdisciplines of sociology, high handbook citation counts are likely to indicate the outstanding, and sometimes canonized, status of scholars within (or even across) different domains of knowledge (e.g., stratification research).

Finally, journals are likely to contain references to cutting-edge scholarship from contemporary scholars whose work has not yet become part of the sociological canon.

I expect high citation counts across different literature genres to indicate “certified recognition” in the discipline, that is, stable and relatively uncontroversial recognition due to outstanding achievement. As three out of the four literature genres are predestined to measure to which degree an author’s contributions have already become widely acknowledged in the literature, the text corpus clearly discriminates against current or rising “stars” in sociology. This is intentional, as the future reception of these scholars is unpredictable and their “elite status” has not yet become uncontroversial.

### Register of Eminent Sociologists

Before turning to citation analyses, I had to introduce a convention on whom to count as a sociologist and how to delimit the number of author names to be searched in the text corpus. I decided to avoid any narrow (and rather arbitrary) definition by taking a performative perspective. I consider scholars as members of the discipline if they have contributed to its core corpus of literature, even if these scholars might not call themselves sociologists (Fleck 2011). Additionally, I limit the present study to living authors and authors who died after (or during) World War I, which leads me to disregard early theorists such as Auguste Comte, Karl Marx, or Herbert Spencer.

As non-selective biographical dictionaries of major sociologists are not available, I had to take an iterative approach and refine results through iterative search processes. First I assembled all available published rosters of eminent sociologists (e.g., Bain 1962; Cronin, Snyder, and Atkins 1997; Oromaner 1980) as well as all rankings of leading scholars in the social sciences by number of citations in the SSCI. Next I checked the bibliographies of all included text materials for author names that appeared frequently and registered all biographical entries in dictionaries and encyclopedias of sociology (e.g., Ritzer 2007). The final register contains 346 authors (see Appendix B).

### Research Strategy

Data were collected from bibliographies (and not the body of the text) without the use of automatized retrieval routines.^9^ Depending on the style of referencing, I also considered footnotes or endnotes. All referenced works were added to the present database. I only registered one citation for any specific work per text source (e.g., journal article or encyclopedia entry) even if the given book, book chapter, or journal article was cited several times in the text.

In contrast to the Institute of Scientific Information, I also weighted the data in consideration of multi-authorship. Single authors were given one point, two joint authors half a point each, three authors a third of a point each, and so on.^10^ In the case of monographs, I did not differentiate between authorship and editorship. To give an example: The editors of “From Max Weber. Essays in Sociology” Hans Gerth and Charles W. Mills are each given a point (in the unweighted search) or a half a point (in the weighted search), even if they only contributed the foreword.

I decided to include self-citations and neglect reference sections of biographical encyclopedia entries (as they would heavily bias the end results). Further, I coded a dichotomous variable indicating whether an author appears in the bibliography of a given contribution or not. Such a measure levels out the tendency of some contributors to extensively draw on work from few authors (or their own work).

Adding up citation counts across sources from an unbalanced text corpus would give unproportioned weight to encyclopedias containing considerably more references than other text sources (e.g., journals). Therefore, I normalized citation scores *x* for all scholars *i* separately for each literature genre (*g*) using the formula =$y_{ig}=\frac{x_{ig}-\min\;\left(x_{g}\right)}{\max\;\left(x_{g}\right)-\min\;\left(x_{g}\right)}.$ The range for citation counts within each literature genre (e.g., handbooks) thus varies between 0 and 1, while citation counts aggregated across all four literature genres vary between 0 and 4. I decided to use this min-max normalization rather than the more common *z*-normalization technique, as positive values are better suited for visualization purposes.

### Biographical Information

To provide a description of those scholars identified as belonging to the prestige elite in the 1970s and 2010s, I gathered information on selected variables that allow to document change in those who comprise the elite across time. The following biographical encyclopedias were among the main search sources: *American National Biography,* Marquis *Who’s Who in America* online biographies of 50 classics in sociology administered by the “Archive for the History of Sociology in Austria”,^11^ the *Dictionary of Modern American Philosophers*, and biographical entries in the *International Encyclopedia of the Social and Behavioral Sciences* (Wright 2015). I also relied on biographical monographs (e.g., Heer 2005), biographical memoirs edited by the National Academy of Sciences (e.g., Scott and Craig 2004), and autobiographical essays (e.g., Sassen 2005).

The variables coded for a collective portrait of the elite are the occupational status of fathers; country of birth; country of residence, migrant status; university from which the elite member received a PhD; university affiliations (full professorships only); presidency of the American Sociological Association (ASA); Guggenheim fellowship and fellowships at different Institutes for Advanced Studies^12^; prestigious awards won, and memberships in honorific societies.^13^

It is perhaps pertinent to offer some illustrations of how these variables were coded. The Spanish-born M. Castells escaped from Franco’s dictatorship to Paris and obtained a PhD in sociology from the Université de Paris in 1967 (he had already obtained a doctorate from the University of Madrid).^14^ He held the following positions: Assistant Professor, University of Paris (1967–1969); Assistant Professor, University of Montreal (1969–1970); Associate Professor, École des Hautes Études en Sciences Sociales (1970–1979); Professor, University of California, Berkeley (1979–2003); Professor, University of Southern California (2003–). The succession of professorships through which Castells moved shows that he spent most of his working life in the United States. All five academic institutions that hosted Castells as a full professor (i.e., University of California, Berkeley; University of Southern California) are registered and considered when analyzing university affiliations. I consider Castells a migrant because he was born and raised in Spain and moved to the United States. In the present definition, being a migrant implies that someone relocated his entire life permanently, which, for example, does not hold true for the Munich-based Ulrich Beck, who was merely an inveterate traveler holding a visiting post at the London School of Economics. In an interview, Castells indicates that both of his parents were civil servants with the Spanish Ministry of Finance; his father was a finance inspector (Castells and Ince 2003:7). In the international and historical HISCO occupation classification (Leeuwen, Maas, and Miles 2002), the code that fits best is “Auditor (1–10.20),” which belongs to the minor group of “Accountants (1–1)” and the major group of “professional, technical and related workers (0/1).” HISCO integrates about 1000 occupational titles and is a highly differentiated international classification. Occupations are classified by economic sector and workplace tasks. Workers in the HISCO major group 0/1, for example, “conduct research and apply scientific knowledge to the solution of a variety of technological, economic, social and industrial problems and perform other professional, technical, artistic and related functions in such fields as the physical and natural sciences, engineering, law, medicine, religion, education, literature, art, entertainment and sport.”^15^

### Reception in the “Journal World”

To reconstruct how an author was received by different audiences I take further cues from scientometrics and focus on the world of academic journals only. The present raw data on citations in academic journals come from Clarivate Analytics’ Web of Science (WoS) which hosts the Social Science Citation Index (1956–).^16^ As WoS is not a full-text database, I worked with reference lists of articles only, thus pursuing similar search strategies as with my own text corpus. WoS allows “cited author” names to be searched for in reference sections using Boolean operators which, for example, would allow James S. Coleman to be searched for using the following variants of the cited author’s name: “Coleman J S or Coleman JS or Coleman James S or Coleman James Samuel.” WoS further allows to systematically export key information on all identified journal articles–such as, for example, the journal’s name (SO) or the journal article’s title (TI). In my analysis, I use these exportable metadata files.

As my interest was to explore whether reception occurs either across different countries and fields of specialization or is confined to single countries (i.e., the United States) or research communities (i.e., political sociology), I counted articles in 18 generalist, 36 specialist, and 30 international sociology journals (see Appendix C) between 1956 and the present that cite a sampled author. While most generalist and specialist journals are published in the United States, all international journals are edited outside of the United States and often feature non-English articles.

## Results

### Toward a Roster of the Most Cited Scholars in Sociology

**Figure 1.**
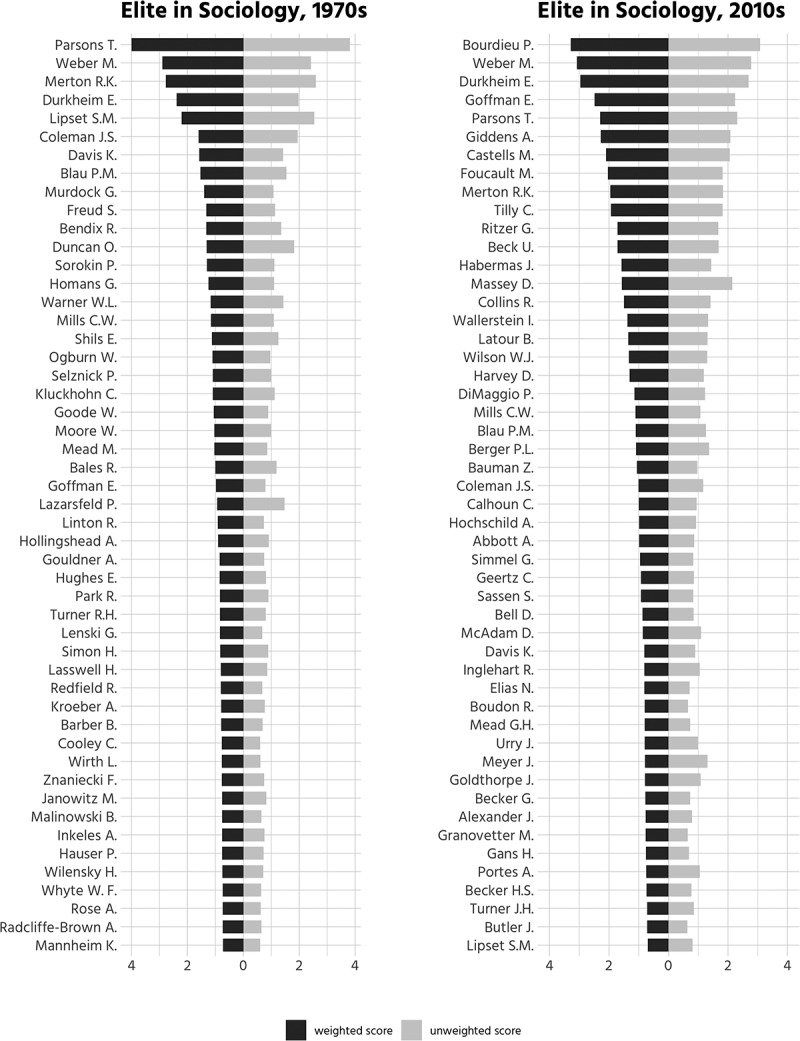
Top 50 sociologists, according to aggregate weighted citation scores (normalized score).

To identify the prestige elite, I ordered all sociologists by citation scores aggregated across four different literature genres (encyclopedias, handbooks, journals, textbooks). Figure 1 suggests that T. Parsons was by far the most eminent sociologist in the 1970s. M. Weber and R. Merton rank second and third and are closely followed by E. Durkheim and S. Lipset, J. Coleman, K. Davis, and P. Blau with similarly outstanding citation scores. The ordering of these top-ranked scholars changes slightly if unweighted citation data are considered. Disregarding coauthors, S. Lipset ranks second and R. Merton third. Additionally, O. Duncan and P. Lazarsfeld, who are both known for empirical contributions written jointly with collaborators, enter the uppermost ranks.

In the 2010s, P. Bourdieu, E. Goffman, A. Giddens, M. Castells, M. Foucault, and C. Tilly join the small group of highly influential scholars. It is, once again, noteworthy that while some scholars—D. Massey, for example—who coauthored many articles, rank slightly higher when citations are not weighted, the lists of top scholars in 2010 based on weighted and unweighted aggregate scores are very similar.

The overall picture that emerges from Figure 1 is a citation distribution that is heavily skewed at the very top and marked by low “prestige gaps” between authors in other parts of the distribution. While the number of authors with high weighted citation scores (>2) grew between 1970s and 2010s, only two authors born in the 20th century remain in top ranks (>10) between both time periods; T. Parsons and R. Merton.

**Figure 2.**
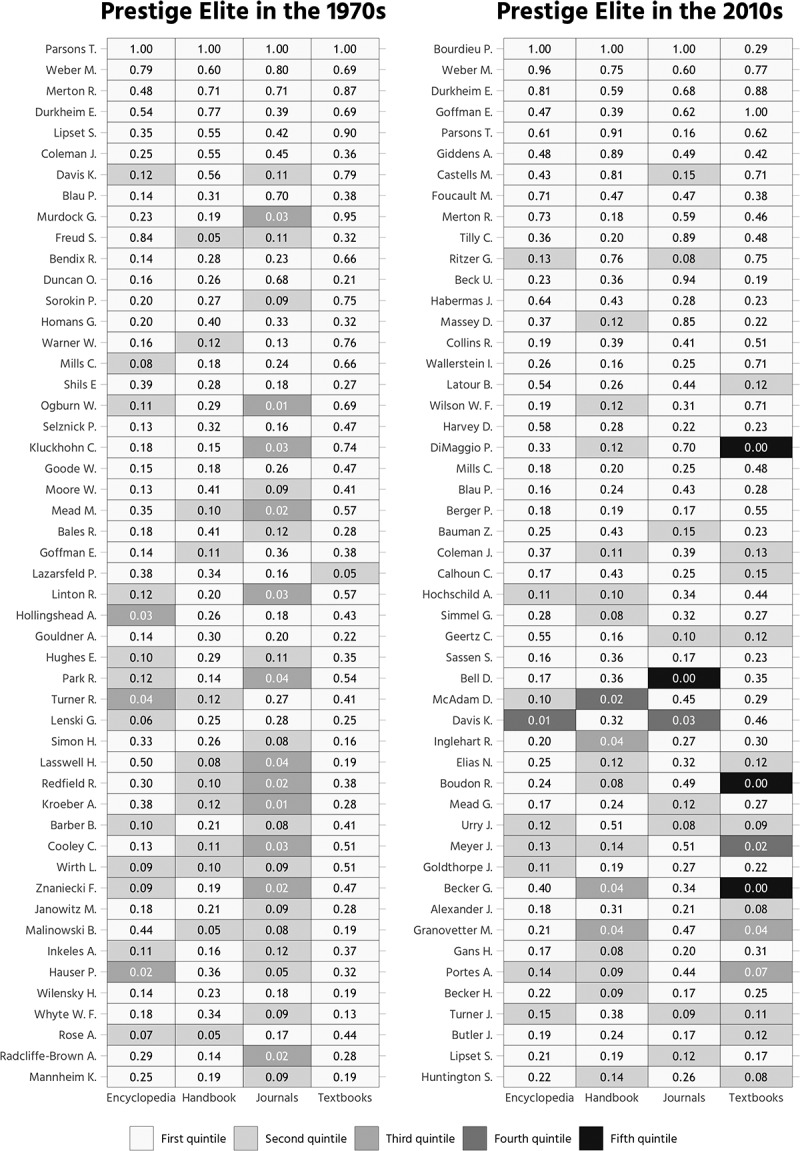
Top 50 sociologists, weighted aggregate citation score decomposed by literature genre. *Note*. Quintiles were calculated separately for each literature genre.

In Figure 2, the aggregate score is decomposed by literature genre. All scores are assigned to text source-specific quintiles. To give an example: If the four different literature genres are analyzed, T. Parsons ranks first in each separate analysis and thus always occupies a rank situated in the first quintile of the four citation distributions analyzed. K. Davis, on the other hand, shows two (of four) citation scores that are assigned to the second quintile (“top 40”) in the encyclopedia and journal-specific distribution.

For the year 1970, Figure 2 points to the comparatively low citation scores of anthropologists such as G. Murdock, C. Kluckhohn, M. Mead, or A. Kroeber, who belong to the fourth quintile in the journal-specific distribution. This finding suggests that peer-reviewed journal articles tended to promote primarily disciplinary knowledge at a time where non-sociologists exercised considerable impact on the discipline. The opposite can be seen in the case of the *International Encyclopedia of the Social Sciences* (Sills 1968), which covered the state of knowledge in many disciplines, among them anthropology and economics. Key sociologists, such as P. Blau or O. Duncan, are not as frequently cited in the encyclopedia’s 9,000 pages as they are in the *Modern Handbook of Sociology* (Faris 1964).

Further, Figure 2 shows that the number of highly cited sociologists across diverse genres of literature increased between 1970 and 2010. It is also interesting to see that sociologists such as P. DiMaggio or A. Abbott have (not yet) achieved textbook eminence. This finding supports the argument that sociology textbooks feature theoretical perspectives on the discipline that neglect the work of leading contemporary sociologists (Manza, Sauder, and Wright 2010).

**Figure 3.**
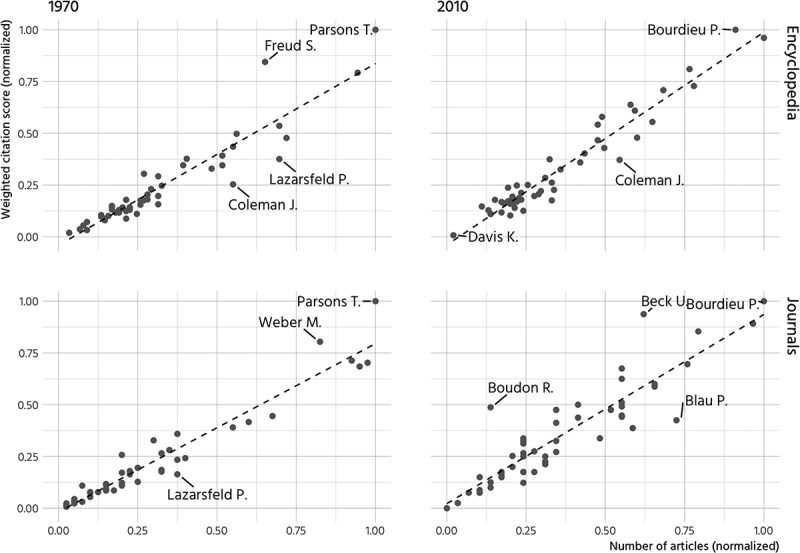
Top 50 sociologists, scatterplot of citation scores and number of articles which reference section contain the scholar’s name.

Citations presented in Figures 1 and 2 are based on research strategies that differ from scholarly impact as measured by Clarivate Analytics. While in both approaches, citations are taken from the reference sections and not the body of the text, I count how often authors are referenced while Clarivate Analytics counts each referenced name only once. To check whether these divergent approaches lead to substantial different results, Figure 3 plots weighted citation scores against the number of journal articles and encyclopedia entries in which authors are mentioned.

It turns out that the correlation is close to perfectly linear (see Figure 3). In the analysis of literature from the 1970s, the weighted citation score attributes S. Freud more importance than the alternative measure that ignores multiple references within encyclopedia entries. This finding suggests that Freud is cited multiple times in a relatively small number of encyclopedia entries.

Coleman and Lazarsfeld would rank higher in the journal- and encyclopedia-specific distribution if the number of articles in which they are referenced at least once was considered as the main indicator of influence. Different contributors to journals and encyclopedias refer to their work, which suggests that their contributions have widely diffused within the various branches of social science specializations. In general, these deviations only have a very minor impact on the ranking of the top 50 sociologists in the 1970s and 2010s.

### Awards and Membership in Honorific Societies

**Figure 4.**
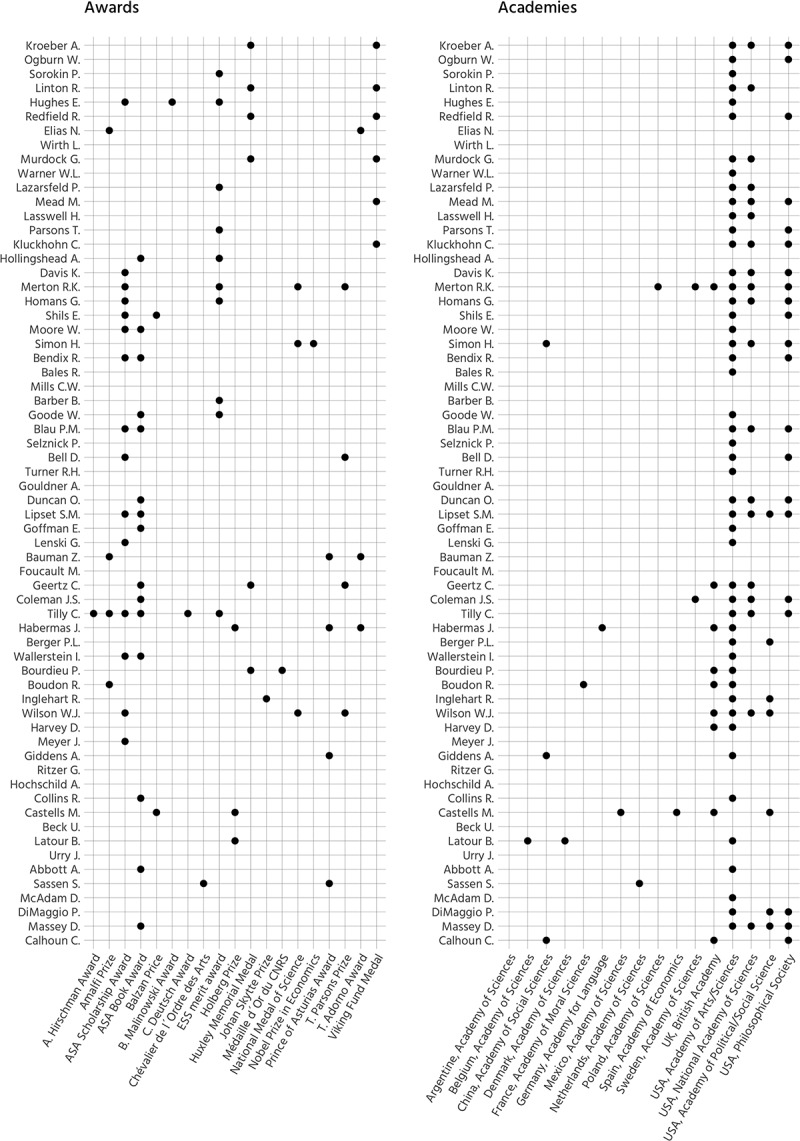
Top 40 sociologists, award received and membership gained in honorific societies (scholars living or having died after 1950). *Note*. Sorted by birth date.

A peculiar feature of top social scientists is that they not only become full professors, but also gain membership of one or more honorific societies and receive prestigious awards (Light, Marsden, and Corl 1973). To check whether this applies to the previously identified top cited social scientists, Figure 4 visualizes to what degree selected top scholars received peer recognition using these two indicators.

It can be inferred that the majority of all selected scholars received at least one prestigious award and were elected into at least one Academy. If one simply considers awards, then Charles Tilly appears to be the most distinguished contemporary sociologist, while Robert K. Merton gained the most memberships in in honorific societies.

A few scholars such as L. Wirth, C. Mills, A. Gouldner, M. Foucault, G. Ritzer, or A. Hochschild received no formal recognition for their achievements. It is difficult, if not impossible, to provide a thorough answer as to why this is the case. Some possible explanations are as follows: L. Wirth and M. Foucault died at a young age, G. Ritzer and A. Hochschild retired only recently, and C. Mills and A. Gouldner had real struggles with the sociological establishment (Chriss 2015). Further, none of these scholars mentored students that became important adherents or developers of their mentor’s ideas. Future awards for some of these scholars are, however, not inconceivable.

It should also be noted that a recipient of the Almalfi, Balzan, and Holberg Prizes—S. Eisenstadt—does not rank among the top cited scholars. Eisenstadt is commonly acknowledged to be a “sociological giant” (Robertson 2011) and his prolific work touches on many different fields of sociology. However, in my 2010 citation ranking, Eisenstadt only ranks 84. There could be various factors at play that cause this comparatively low citation score, one being the fact that Eisenstadt’s most outstanding macrosociological work on the political systems of empires reached only small academic audiences.

### The Changing Elite Composition between 1970 and 2010

What are the characteristics shared by elite groups in sociology and how did they change between the 1970s and 2010s? To derive a collective portrait, simple cross-tabulations are applied in Table 2.

**Table 2.** Social characteristics of the elites in the 1970s and 2010s (scholars living or having died after 1950).

	1970 (%)	2010 (%)
*Father’s occupation (HISCO major group)*		
Professional, Technical and Related Workers (0/1)	32.6	30.4
Administrative and Managerial Workers (2)	4.7	17.4
Clerical and Related Workers (3)	4.7	6.5
Sales Workers (4)	20.9	17.4
Service Workers (5)	2.3	2.2
Agricultural, Animal Husbandry and Forestry Workers (6)	9.3	4.3
Production and Related Workers (7/8/9)	11.6	15.2
No information available	14.0	6.5
*Male*	97.7	93.5
*Migrant*	16.3	21.7
*Country of birth*		
Austria	4.7	4.3
Canada	2.3	2.2
Cuba		2.2
France		8.7
Germany	4.7	8.7
Netherlands		2.2
Poland	2.3	2.2
Russia	2.3	
Spain		2.2
United Kingdom	2.3	8.7
United States	81.4	58.7
*Country of residence*		
France		8.7
Germany		4.3
United Kingdom	2.3	10.9
United States	97.7	76.1
*Main disciplinary background*		
Anthropology	18.6	2.2
Economics	2.3	2.2
Geography		2.2
Philosophy		6.5
Political science	4.7	2.2
Sociology	74.4	84.8
*ASA President^a^*	46.5	26.1
*Awarded Guggenheim Fellowship^b^*	35.7	41.3
*Institution granting PhD*		
Cambridge University	2.3	4.4
Columbia University	20.9	13.0
Cornell University		4.3
École Normale Supérieure		4.3
Harvard University	20.9	13.0
University of California, Berkeley		6.5
University of Chicago	34.9	10.9
University of Wisconsin–Madison		4.3
Yale University	4.7	2.2
Other universities	16.3	41,4
*Research fellowship location*		
Center for Advanced Study in the Behavioral Sciences	41.9	45.7
Institute for Advanced Study/Princeton	4.7	17.4
Institute for Advanced Study/Berlin	2.3	6.5
Netherlands Institute for Advanced Study	4.7	8.7
Swedish Collegium		8.7
*Full professorships at*		
Cambridge University		2.0
Columbia University	10.4	10.1
École des Hautes Études en Sciences Sociales		1.0
Harvard University	10.4	4.0
John Hopkins University	1.3	5.1
London School of Economics		2.0
Oxford University	1.3	2.0
Princeton University	1.3	4.0
Stanford University	3.9	4.0
Universität Frankfurt		2.0
Université de Paris		2.0
University at Michigan, Ann Arbor	3.9	2.0
University of California, Berkeley	9.1	6.1
University of Chicago	14.3	8.1
University of Pennsylvania	1.3	3.0
University of Wisconsin–Madison	1.3	
Yale University	5.2	2.0
Other universities	36.3	40.6
*Number of full professorships per scholar*		
No professorship	2.3	
1 professorship	46.5	37.5
2 professorships	30.2	25.0
3 professorships	11.6	12.5
4 professorships	9.3	25.0
*Number of scholars considered*	43	46

^a^If one considers U.S. residents only, then the numbers change to 47.6% and 34.3%.

^b^If one considers U.S. residents only, then the numbers change to 36.6% and 54.3%.

The picture that emerges from Table 2 is one of an American-dominated elite in the 1970s that became considerably more Europeanized by the 2010s. The number of presidents of American-dominated professional organizations (ASA) decreases over time. While the top sociologists of the 1970s mostly received their PhD from Columbia University, Harvard University, or University of Chicago, such a unique breeding ground no longer exists for contemporary leading scholars. Similarly, one can observe that in 2010 the elite was substantially more scattered across various universities. In general, elite institutions such as Cambridge University or EHESS host more elite members than average academic institutions do. Between 1970 and 2010 the academic elite has become more mobile with about one quarter of elite members switching professorships up to four times, which suggests, among other things, that scholars have become more able to leave their posts and take their talents elsewhere (perhaps for better remunerations or a reduction in administrative duties).

Interestingly, anthropologists are included in the 1970 elite and philosophers—such as Jürgen Habermas and Judith Butler—joined top ranks in the 2010s. The share of female scholars remains below 5% in both years. Across time, one can observe that elites benefit from sponsorship systems promoting academic excellence; the proportion of scholars awarded the prestigious Guggenheim Foundation fellowship is about 40%.

Another career contingency is that academic elites tend to work temporarily at Institutes for Advanced Studies where they are surrounded by researchers rather than students and are therefore freed from the usual faculty commitments. The Center for Advanced Study in the Behavioral Sciences appears to be most important intellectual hub. While it is impossible to unambiguously determine the social origin of the academic elite, in part because of some missing data on paternal occupation, it nevertheless transpires from Table 2 that the background is clearly more the “educated upper middle class” (e.g., jurists or university professors) than the “economic upper middle class” (e.g., managers). However, a substantial proportion of elite members also had fathers with commercial or blue-collar occupations. Over time, the number of scholars in the HISCO major group 6–9 (farmers and workers) remains at about 20%, which suggests that the prestige elites in sociology continue to be open to diverse backgrounds.

### An Analysis of the Space of Reception

**Figure 5.**
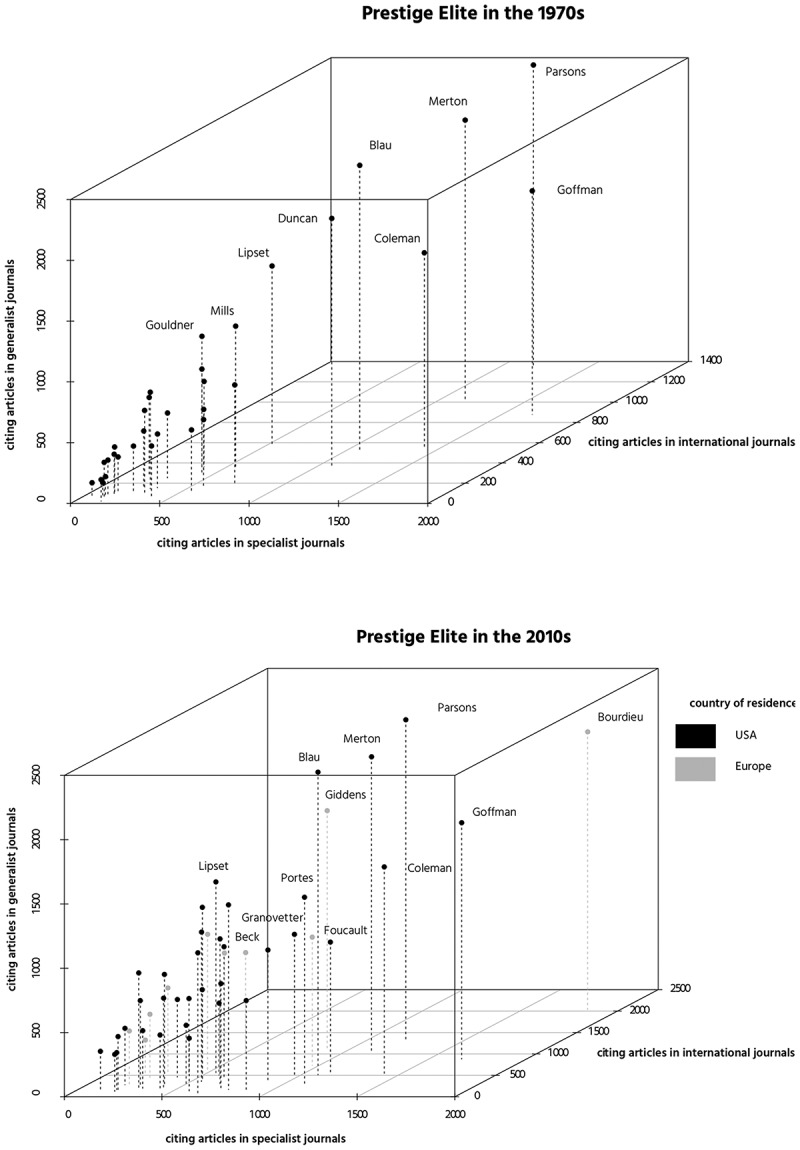
The space of reception in academic journals (scholars born after 1900). *Notes*. Both panels are derived from the same Web of Science dataset that includes 74,339 articles published between 1956 and 2018 in 36 specialist, 18 generalist, and 30 international academic journals (see Appendix C). The coordinates of P. Bourdieu (2032;1834;2162) stand for 2,032 articles in international, 1,834 articles in specialist, and 2,162 articles in generalist journals that cite Bourdieu. The range of the x-axis changes between both panels because of Bourdieu’s outstanding high values on the international dimension.

To uncover which groups of scholars are influenced by the diverse contributions of prestige elites I construct a three-dimensional space that allows simultaneous study of the impact of all elite members on three types of (past and present) academic audiences: generalists, specialists, and sociologists outside the United States. Figure 5 allows for a comparison of members of both the 1970s and 2010s prestige elites with each other. In the 1970s elite, for example, E. Goffman is more often cited in specialist journals than T. Parsons, but in generalist and international journals his ideas are less often borrowed. As both panels in Figure 5 are derived from the same dataset, the same pattern becomes visible for the 1970s and 2010s elite (as E. Goffman and T. Parsons are among the few scholars who stay top ranked over time).

In the 1970s elite, a small group of scholars including T. Parsons, R. K. Merton, E. Goffman, J. S. Coleman, P. M. Blau, and O. Duncan succeeds in deeply impacting all three audiences. All other scholars have substantially less impact if one considers the world of academic journals. Some scholars, such as S. M. Lipset, were better received by general audiences than by highly specialized audiences.

The picture changes for the 2010s elite in which P. Bourdieu trumps R.K. Merton and even T. Parsons on all dimensions except the generalist one. With A. Giddens and M. Foucault two other Europeans join the high-impact group of scholars. It is interesting to see, once again, that no scholar scores higher on the specialist than on the generalist dimension, which suggests that the reputation of elite sociologists is not confined to single specialized areas of the discipline.

Overall, it transpires that there is a deep inequality in reception when it comes to academic journals and that eminence rankings derived exclusively from references appended to journal articles produce a very different picture of scholarly impact than rankings based on references in monographic literature (Cronin, Snyder, and Atkins 1997). If, for example, one based the rankings solely on journal articles, none of the sampled anthropologists (see Figure 1) would make it into the roster of the top 50.

## Discussion

Based on a text corpus encompassing about 49,000 pages, this article identified prestige elites by relying on citation scores. Where the SSCI covers academic journals only, I analyzed a text corpus incorporating encyclopedias, handbooks, and textbooks, thus giving substantial weight to monographic literature, which is more cited in these genres of literature than in the “journal world.” Such a broad approach appeared to better fit the discipline as previous explorations of citation data revealed that sociologists “attend to and cite leading books at even higher rates than they cite leading articles” (Sullivan 1994:171). Moreover, citations in the monographic literature are better manifestations of “*certified recognition*” than journal article citations are.

A recurrent criticism of citation studies in eminence research is that the meaning of citations is unclear. I show that, with the notable exception of S. Eisenstadt, very high citation levels correlate with honorific awards and membership of honorific societies. Therefore, in the case of the prestige elite, citations can be used as an approximate indicator of influence as they reflect evaluations of scientific contributions by qualified peers. As I show, the ranking of social scientists changes only slightly according to the weight given to coauthors.

Apart from considering various genres of literature, the analysis focused on citations across time. The results suggest that, in general, scholarly eminence has a short life-span (<40 years). Besides E. Durkheim and M. Weber, only two 20th century scholars—R. Merton and T. Parsons—remained among the top 10 scholars between 1970 and 2010. The waxing and waning of influence becomes apparent with regard to S. Lipset, for example, who was once the “most cited social scientist in the world” (Fischer and Swidler 2016:2).

First investigations revealed that the prestige elite has become more European and less American over time. The finding that most top sociologists received their PhD from Columbia University, Harvard or Chicago University in the 1930s is compatible with the observation that sociology departments at these universities were key in establishing disciplinary norms (Turner and Turner 1990). Less than 20% of the 1970s eminent scholars, such as P. Blau, R. Bendix, or P. Sorokin, migrated from Austria, Germany, and Russia to the United States. Increased heterogeneity of the elite in the 2010s can be explained by the fact that sociology is currently firmly established as a discipline in almost every university.

The analysis might, to some degree, overestimate both the homogeneity of the 1970s elite and the formative role of American institutions in the education of future star sociologists. Back in the 1970s encyclopedias (and handbooks) were only international in a limited sense, which attracted much criticism. But even critics such as MacLeod in his review article of the considered 1970 encyclopedia (Sills 1968) concluded that “contemporary social science *is* predominantly American … Blame it on the affluent society, or on an academic system that rewards sheer quantity of publication, or even on the availability of a Xerox machine; the fact remains that the English-reading public is being deluged” (MacLeod 1970:714).

Another major finding is the continuing social openness of the elite between 1970 and 2010. While about one third of all scholars have educated upper-class background such “social climbers” as Seymour M. Lipset or Pierre Bourdieu are by no means exceptions, but reflect the heterogeneity of the elite’s social origins. Much suggests that such absence of a “class wall” stands in stark contrast to the social closure of academia in other disciplines such as economics (Lebaron 2006).

Explorations of reception patterns inside sociology’s “journal world” convey the picture of a highly unequal reception: Only very few scholars such as P. Bourdieu, R. K. Merton, or A. Giddens succeed in deeply impacting international, specialist, and general audiences. The ideas of the current “stars” of European sociology, among them U. Beck, circulate today to the same extent within the United States dominated mainstream sociology as, for example, those of the American sociologist M. Granovetter (Ollion and Abbott 2016). In general, the identified reception patterns reveal that elite sociologists are always well received not only within subcommunities of the discipline but also within the broad core of the discipline.

### Limitations and Suggestions for Future Research

An obvious limitation of this study is focus on selected monographic literature such as handbooks, textbooks and encyclopedias. However, as I have made my dataset available (see the Supplementary Material), other scholars may decide to systematically extend the text corpus by including citations in other representative monographs (and leading journals). The study of Cronin, Snyder, and Atkins (1997) might provide orientation on how to build a database of monographic citations from a random sample of books.

In future, perhaps the role of Guggenheim foundation fellowships and research stays at Institutes for Advanced Studies such as the Center for Advanced Study in the Behavioral Science at Stanford University might be analyzed in more depth. This study reveals only that both mark career stages through which members of the prestige elite in sociology commonly move. Further, this study proves that a “glass ceiling” exists for female academics with regard to sociology’s prestige elite. Clearly, additional research is needed to explain why women are underrepresented in the highest prestige strata of the discipline. Finally, the profile of prestige elites could be better worked out in a comparative framework that additionally considers, for example, average scholars in sociology or prestige elites in other social science disciplines. Given the limited space given to journal articles, this study was unable to include relevant comparison groups.

## Notes

1. Please find the article’s dataset as well as the data codebook in the Supplemental Material.

2. In his massive *Treatise on General Sociology* first published in 1916, Pareto suggested a highly formal elite definition: “Let us assume that in every branch of human activity each individual is given an index which stands as a sign of his capacity, very much the way grades are given in the various subjects in examinations in school. The highest type of lawyer, for instance, will be given 10. The man who does not get a client will be given 1—reserving zero for the man who is an out-and-out idiot … So let us make a class of the people who have the highest indices in their branch of activity, and to that class give the name … of élite” (Pareto 1935:1423).

3. Presidents of the ASA occupy a significant administrative position. However, whether or not these presidents are marked only by executive power, or whether they also have an outstanding scholarly impact is a question that can only be answered empirically (Platt 2016).

4. When asking for peer evaluation, Cattell wanted judges to consider multiple criteria: “… primarily by research, but teaching, administration, editing, the compilation of textbooks, etc., should be considered…The men should be ranked for work actually accomplished, that is, a man of sixty and a man of forty, having done about the same amount of work, should come near together, though the man of forty has more promise” (Cattell 1906:660).

5. Historical evidence suggests that the complexity of the task at hand usually leads to inconsistent conceptions of what it means to be a leading scholar. Even if Cattell spelled out different evaluation criteria (see endnote 4), some surveyed members of the National Academy of Science tended to give more weight to publication records while others made an appraisal considering various other criteria of scholarly recognition (Meltzer 2002).

6. In a more recent citation study on 57 Nobel Prize winners in economics, and in a similar vein, Bjork et al. (2014) found that the Swedish Academy tends to award the prize closer to the winners’ citation peak (i.e., at the height of the winners’ reputations).

7. One should add that at least in American sociology, the 1970s marked an important turning point: Parsonianism in its orthodox form did not survive Gouldner’s *Coming Crisis of Western Sociology* of 1970 and the financial resources for sociology had begun to collapse (Turner 2014).

8. I excluded from my analysis contributions in journals that do not follow the usual standards of referencing such as, for example, notes of journal editors, obituaries, or book reviews.

9. There are two main reasons why I decided not to apply automatized retrieval routines. First, referenced names (e.g., Hauser, Wright) may refer to different individuals. Second, the reference styles differ hugely across time and text sources, which makes even flexible reference search strategies error-prone. In general, working with bibliographies and not citations in the main text allowed to easily verify the author’s identity.

10. The “degree of parallelization” is, of course, difficult to determine, and dividing by the number of authors (1/n rule) is only one possible solution (de Mesnard 2017). The chosen approach, like all other available technical solutions, attributes credit to the substantive contributions of the coauthors.

11. To systematically verify presidencies and fellowships official homepages/search engines of the ASA and of the Guggenheim Memorial Foundation were used. The Institute for Advanced Studies at Princeton University, the Center for Advanced Study in the Behavioral Sciences at Stanford University, the Swedish Collegium for Advanced Study, the Wissenschaftskolleg zu Berlin, and the Netherlands Institute for Advanced Study list all past and present fellows online.

12. Regarding academic awards and memberships in Academies, I did not rely only on self-reports or biographical information, but rather tried, wherever possible, to cross-verify information. Information on recipients of awards are always public and most Academies provide registers of active and past Academy members.

13. Manuel Castells gives in self-reports more weight to the PhD gained in France that dealt with location strategies on industrial firms in the Paris region and paved his career as one of the founders of the “New Urban Sociology” (Castells and Ince 2003). I thus registered the Université de Paris as the PhD granting institution.

14. For a detailed description of all HISCO major groups see: https://collab.iisg.nl/web/hisco/hiscotree, accessed December 10, 2018.

15. To be found here: http://www.webofknowledge.com.

16. It should be noted, however, that some of these researchers received awards that are not included in Figure 4. Arlie Hochschild, for example, received the Jessie Bernard Award in 2008.

## APPENDIX A

### 1970

#### Textbooks considered

Bierstedt, Robert (1974) *The Social Order: An Introduction to Sociology*. New York: McGraw-Hill.

Broom, Leonard and Philip Selznick (1973) *Principles of Sociology. A Text with Adapted Readings*. New York: Harper & Row.

Horton, Paul B. and Chester L. Hunt (1964) *Sociology*. New York: McGraw-Hill.

Inkeles, Alex (1964) *What is Sociology? An Introduction to the Discipline and Profession*. Englewood Cliffs, New Jersey: Prentice-Hall.

Lenski, Gerhard (1970) *Human Societies: A Macrolevel Introduction to Society*. New York: McGraw-Hill.

#### Handbook considered

Faris, Robert E., ed. (1964) *Handbook of Modern Sociology*. Chicago: Rand McNally.

#### Encyclopedia considered

Sills, David L. ed. (1968) *International Encyclopedia of the Social Sciences*. New York: The Macmillan Company and the Free Press.

### 2010

#### Textbooks considered

Giddens, Anthony and Philip W. Sutton (2009) *Sociology*. Cambridge: Polity Press.

Henslin, James M. (2015) *Essentials of Sociology: A Down-To-Earth Approach*. Boston: Pearson.

Kendall, Diana E. (2011) *Sociology in Our Times*. Belmont, CA: Wadsworth/Cengage Learning.

Macionis, John J. (2012) *Sociology*. Boston: Pearson.

Schaefer, Richard T. (2013) *Sociology: A Brief Introduction*. Dubuque, Iowa: McGraw-Hill.

#### Handbook considered

Calhoun, Craig J., Chris Rojek and Bryan S. Turner, ed. (2005) *The Sage Handbook of Sociology*. London: Sage Publications.

#### Encyclopedia considered

Wright, James D., ed. (2015) *International Encyclopedia of the Social & Behavioral Sciences*. Amsterdam: Elsevier.

## APPENDIX B

**Table:**

*First Name*		*Second Name*	*Born*	*Died*
Alfred		Marshall	1842	1924
Granville S.		Hall	1846	1924
John B.		Clark	1847	1938
Vilfredo		Pareto	1848	1923
Albion W.		Small	1854	1926
Franklin H.		Giddings	1855	1931
Ferdinand		Tönnies	1855	1936
Sigmund		Freud	1856	1939
Emil W.		Kraepelin	1856	1926
Thorstein		Veblen	1857	1929
Franz		Boas	1858	1942
Émile		Durkheim	1858	1917
Gaetano		Mosca	1858	1941
Georg		Simmel	1858	1918
Beatrice		Webb	1858	1943
John		Dewey	1859	1952
Edmund G.		Husserl	1859	1938
George H.		Mead	1863	1931
Werner		Sombart	1863	1941
William I.		Thomas	1863	1947
Charles H.		Cooley	1864	1929
Robert E.		Park	1864	1944
Max		Weber	1864	1920
Irving		Fisher	1867	1947
W.E.B.		Du Bois	1868	1963
Marcel		Mauss	1872	1950
Max		Scheler	1874	1928
Carl G.		Jung	1875	1961
Ellsworth		Huntington	1876	1947
Alfred		Kroeber	1876	1960
Robert		Michels	1876	1936
Maurice		Halbwachs	1877	1945
Alfred R.		Radcliffe-Brown	1881	1955
Robert M.		MacIver	1882	1970
Florian		Znaniecki	1882	1958
John		Keynes	1883	1946
Joseph		Schumpeter	1883	1950
Edwin		Sutherland	1883	1950
Bronislaw		Malinowski	1884	1942
Howard W.		Odum	1884	1954
Edward		Sapir	1884	1939
Marc		Bloch	1886	1944
Ernest W.		Burgess	1886	1966
Helen M.		Lynd	1886	1982
William F.		Ogburn	1886	1959
Karl		Polanyi	1886	1964
Ruth		Benedict	1887	1948
Warren S.		Thompson	1887	1973
Carl		Schmitt	1888	1985
Jacob L.		Moreno	1889	1974
Pitrim A.		Sorokin	1889	1968
Ludwig		Wittgenstein	1889	1951
Kurt		Lewin	1890	1947
Theodor		Geiger	1891	1952
Vere G.		Childe	1892	1957
Ralph		Linton	1893	1953
Karl N.		Llewellyn	1893	1962
Robert S.		Lynd	1892	1970
Karl		Mannheim	1893	1947
Thomas H.		Marshall	1893	1981
Alfred C.		Kinsey	1894	1956
Jerzy		Neyman	1894	1981
Harold		Hotelling	1895	1973
Max		Horkheimer	1895	1973
George A.		Lundberg	1895	1966
Lewis		Mumford	1895	1990
William		Kornhauser	1896	1990
Sheldon		Glueck	1896	1980
Fritz		Heider	1896	1988
Roman O.		Jakobson	1896	1982
Jean		Piaget	1896	1980
Lev S.		Wygotski	1896	1934
Gordon		Allport	1897	1967
Norbert		Elias	1897	1990
Everett C.		Hughes	1897	1983
George P.		Murdock	1897	1985
Robert		Redfield	1897	1958
Louis		Wirth	1897	1952
Herbert		Marcuse	1898	1979
Gunnar		Myrdal	1898	1987
Fritz J.		Roethlisberger	1898	1974
William L.		Warner	1898	1970
Alfred		Schütz	1899	1959
Dorothy S.		Thomas	1899	1977
Willard W.		Waller	1899	1945
Herbert		Blumer	1900	1987
Erich		Fromm	1900	1980
Samuel A.		Stouffer	1900	1960
Leslie A.		White	1900	1975
René J.		Dubos	1901	1982
Paul		Lazarsfeld	1901	1976
Margaret		Mead	1901	1978
Erik H.		Erikson	1902	1994
Edward E.		Evans-Pritchard	1902	1973
Harold D.		Lasswell	1902	1978
Oskar		Morgenstern	1902	1977
Alva		Myrdal	1902	1986
Talcott		Parsons	1902	1979
Karl R.		Popper	1902	1994
Theodor W.		Adorno	1903	1969
Jessie		Bernard	1903	1996
Konrad Z.		Lorenz	1903	1989
George E.		Simpson	1904	1991
Burrhus F.		Skinner	1904	1990
Raymond C.		Aron	1905	1983
Carl G.		Hempel	1905	1997
Clyde K.		Kluckhohn	1905	1960
Mirra		Komarovsky	1905	1999
Hannah		Arendt	1906	1975
Hadley		Cantril	1906	1969
Muzafer		Sherif	1906	1988
John		Bowlby	1907	1990
Mircea		Eliade	1907	1986
Robert E.		Faris	1907	1998
August B.		Hollingshead	1907	1980
Marie		Jahoda	1907	2001
Kingsley		Davis	1908	1997
Hans H.		Gerth	1908	1978
Claude		Lévi-Strauss	1908	2009
Peter F.		Drucker	1909	2005
Philip M.		Hauser	1909	1994
David		Riesman	1909	2002
William H.		Sewell	1909	2001
Ester		Boserup	1910	1999
George C.		Homans	1910	1989
Robert K.		Merton	1910	2003
Edward A.		Shils	1910	1995
Leonard S.		Broom	1911	2009
Matilda W.		Riley	1911	2004
George J.		Stigler	1911	1991
Bernard R.		Berelson	1912	1979
Karl W.		Deutsch	1912	1992
Edwin M.		Lemert	1912	1996
Lewis A.		Coser	1913	2003
Leonard S.		Cottrell	1913	1974
Walter		Goldschmidt	1913	2010
Bert F.		Hoselitz	1913	1995
Delbert C.		Miller	1913	1998
Barrington		Moore	1913	2005
Robert A.		Nisbet	1913	1996
Paul		Ricoeur	1913	2005
Wilbert E.		Moore	1914	1987
William F.		Whyte	1914	2000
Robin M.		Williams	1914	2006
Roland		Barthes	1915	1980
Jerome S.		Bruner	1915	2016
Dorwin P.		Cartwright	1915	2008
Robert A.		Dahl	1915	2014
Albert O.		Hirschman	1915	2012
Robert F.		Bales	1916	2004
Reinhard		Bendix	1916	1991
Donald T.		Campbell	1916	1996
Jane		Jacobs	1916	2006
Charles W.		Mills	1916	1962
Herbert A.		Simon	1916	2001
Anselm L.		Strauss	1916	1996
William J.		Goode	1917	2003
Bernard		Barber	1918	2006
Morroe		Berger	1918	1981
Peter M.		Blau	1918	2002
Herbert H.		Hyman	1918	1985
Arnold M.		Rose	1918	1968
David M.		Schneider	1918	1995
Daniel		Bell	1919	2011
Leon		Festinger	1919	1989
Morris		Janowitz	1919	1988
Philip		Selznick	1919	2010
Ralph H.		Turner	1919	2014
Thomas B.		Bottomore	1920	1992
Alvin W.		Gouldner	1920	1980
Alex		Inkeles	1920	2010
Albert		Memmi	1920	
David L		Sills	1920	2015
James D.		Thompson	1920	1973
Hans		Albert	1921	
Otis D.		Duncan	1921	2004
Harold		Kelley	1921	2003
John		Rawls	1921	2002
Stein		Rokkan	1921	1979
Peter H.		Rossi	1921	2006
Michel		Crozier	1922	2013
Erving		Goffman	1922	1982
Thomas		Kuhn	1922	1996
Seymour M.		Lipset	1922	2006
Albert J.		Reiss	1922	2006
Alice		Rossi	1922	2009
Stanley		Schachter	1922	1997
Shmuel N		Eisenstadt	1923	2010
Eliot		Freidson	1923	2005
André		Gorz	1923	2007
Harold L.		Wilensky	1923	2001
Kurt		Lang	1924	
Gerhard E.		Lenski	1924	2015
Zygmunt		Bauman	1925	2017
Ernest		Gellner	1925	1995
Alain		Touraine	1925	
Richard A.		Cloward	1926	2001
James S.		Coleman	1926	1995
Michel		Foucault	1926	1984
Clifford J.		Geertz	1926	2006
Raul		Hilberg	1926	2007
Elihu		Katz	1926	
Samuel P.		Huntington	1927	2008
Lawrence		Kohlberg	1927	1987
Niklas		Luhmann	1927	1998
Robert N.		Bellah	1927	2013
Herbert J.		Gans	1927	
Thomas		Luckmann	1927	2016
Charles R.		Wright	1927	2017
Howard S.		Becker	1928	
Noam		Chomsky	1928	
Aaron V.		Cicourel	1928	
James G.		March	1928	2018
Morris		Zelditch	1928	2017
Jean		Baudrillard	1929	2007
Peter L.		Berger	1929	2017
Amitai		Etzioni	1929	
Ralf		Dahrendorf	1929	2009
Jürgen		Habermas	1929	
Alasdair		MacIntyre	1929	
Thomas		Scheff	1929	
Charles		Tilly	1929	2008
Pierre		Bourdieu	1930	2002
Gary S.		Becker	1930	2014
Johan		Galtung	1930	
Barney G.		Glaser	1930	
Neil J.		Smelser	1930	2017
Immanuel		Wallerstein	1930	
Harrison C.		White	1930	
Kai T.		Erikson	1931	
Everett		Rogers	1931	2004
Charles		Taylor	1931	
Mayer N.		Zald	1931	2012
Stuart		Hall	1932	2014
Mancur L.		Olson	1932	1998
William R.		Scott	1932	
John		Searle	1932	
Stanley		Lieberson	1933	
Arthur		Stinchcombe	1933	2018
Raymond		Boudon	1934	2013
William A.		Gamson	1934	
Ronald		Inglehart	1934	
John H.		Goldthorpe	1935	
David W.		Harvey	1935	
John W.		Meyer	1935	
Edward W.		Said	1935	2003
Michael L.		Walzer	1935	
William J.		Wilson	1935	
Michael		Mulkay	1936	
Helga		Nowotny	1937	
Amos		Tversky	1937	1996
Anthony		Giddens	1938	
Edward O.		Laumann	1938	
Sidney G.		Tarrow	1938	
Luc		Boltanski	1940	
Jon		Elster	1940	
Arlies R.		Hochschild	1940	
Claus		Offe	1940	
Orlando		Patterson	1940	
George		Ritzer	1940	
Stephan		Cole	1941	
Randall		Collins	1941	
Jürgen H.		Kocka	1941	
Aage B.		Sørensen	1941	2001
Göran		Therborn	1941	
Manuel		Castells	1942	
Jonathan R.		Cole	1942	
Michael		Mann	1942	
Nico		Stehr	1942	
Jonathan H.		Turner	1942	
Elijah		Anderson	1943	
Margaret S.		Archer	1943	
Harry		Collins	1943	
Mark S.		Granovetter	1943	
Richard		Sennett	1943	
Ulrich		Beck	1944	2015
Colin		Crouch	1944	
Peter		Evans	1944	
Karin		Knorr-Cetina	1944	
Alejandro		Portes	1944	
Ann		Swidler	1944	
Michel		Callon	1945	
Scott		Lash	1945	
Paul		Willis	1945	
John R.		Urry	1946	2016
Stephen W.		Raudenbush	1946	
Wolfgang		Streeck	1946	
Viviana A.		Zelizer	1946	
Jeffrey C.		Alexander	1947	
Michael		Burawoy	1947	
Gøsta		Esping-Andersen	1947	
Bruno		Latour	1947	
Cecilia L.		Ridgeway	1947	
Theda		Skocpol	1947	
Erik O.		Wright	1947	
Andrew		Abbott	1948	
Hans		Joas	1948	
Richard		Swedberg	1948	
Arjun		Appadurai	1949	
Ronald S.		Burt	1949	
Axel		Honneth	1949	
Saskia		Sassen	1949	
John P.		Scott	1949	
Tom A.		Snijders	1949	
Seyla		Benhabib	1950	
Paul J.		DiMaggio	1951	
Neil		Fligstein	1951	
Douglas		McAdam	1951	
Walter W.		Powell	1951	
Stephen P.		Turner	1951	
Craig J.		Calhoun	1952	
Diego		Gambetta	1952	
Douglas		Massey	1952	
Trevor J.		Pinch	1952	
Charles C.		Ragin	1952	
Hans-Peter		Blossfeld	1954	
Bo		Rothstein	1954	
David W.		Garland	1955	
Peter		Hedström	1955	
Judith		Butler	1956	
Frank		Dobbin	1956	
Michèle		Lamont	1957	
Loïc		Wacquant	1960	
Eva		Illouz	1961	
Juliet		Corbin	unkown	
Nancy		Denton	unkown	
Mustafa		Emirbayer	unkown	
Joe R.		Feagin	unkown	
Robert M.		Hauser	unkown	
Juliet	Corbin		unkown	
Nancy	Denton		unkown	
Mustafa	Emirbayer		unkown	
Joe R.	Feagin		unkown	
Robert M.	Hauser		unkown	
James	Mahoney		unkown	
Miller	McPherson		unkown	
Anthony	Orum		unkown	
Mike	Savage		unkown	
Gideon	Sjoberg		unkown	
David A.	Snow		unkown	
Alice	Sullivan		unkown	
Karl E.	Taeuber		unkown	
Brian	Uzzi		unkown	

## APPENDIX C

### Specialist journals in the Web of Science

**Table:**

Subfields of Sociology	Journal
Deviance	*Deviance Behavior*
Ecology	*Human Ecology*
Education	*Sociology of Education*
Ethnography	*Ethnography*
Family	*Journal of Marriage and Family*
Gender	*Gender & Society*
Gender	*Men and Masculinities*
Health	*Journal of Health and Social Behavior*
Health	*Society and Mental Health*
Law	*Journal of Law and Society*
Law	*Law & Society Review*
Leisure	*Journal of Leisure Research*
Mathematical Sociology	*Journal of Mathematical Sociology*
Methodology	*Sociological Methods & Research*
Political, Economic and Work Sociology	*American Journal of Economics and Sociology*
Political, Economic and Work Sociology	*International Political Sociology*
Political, Economic and Work Sociology	*Journal of Political & Military Sociology*
Political, Economic and Work Sociology	*Politics & Society*
Political, Economic and Work Sociology	*Socio-Economic Review*
Political, Economic and Work Sociology	*Work and Occupations*
Political, Economic and Work Sociology	*Work Employment and Society*
Quality of Life	*Social Indicators Research*
Race	*Ethnic and Racial Studies*
Race	*Race and Social Problems*
Rational Choice	*Rationality & Society*
Religion	*Journal for the Scientific Study of Religion*
Religion	*Review of Religious Research*
Rural People/Places	*Rural Sociology*
Social Biology	*Social Biology*
Social Networks	*Social Networks*
Social Stratification	*Research in Social Stratification and Mobility*
Sociology of Culture	*Cultural Sociology*
Sociology of Culture	*Media Culture & Society*
Sociology of Culture	*Poetics*
Sport	*Sociology of Sport Journal*
Youth	*Youth & Society*

### Generalist journals in the Web of Science

**Table:**

*American Journal of Sociology*
*American Sociological Review*
*Annual Review of Sociology*
*British Journal of Sociology*
*Journal of Sociology*
*Pacific Sociological Review*
*Social Forces*
*Social Science Quarterly*
*Social Science Research*
*Sociological Focus*
*Sociological Forum*
*Sociological Inquiry*
*Sociological Perspectives*
*Sociological Quarterly*
*Sociological Spectrum*
*Sociology and Social Research*
*Sociology-The Journal of the British Sociological Association*
*Theory and Society*

### International journals in the Web of Science

**Table:**

Country/Countries	Journal
Australia	*Australian and New Zealand Journal of Sociology*
Canada	*Cahiers Internationaux de Sociologie*
Canada	*Canadian Journal of Sociology—Cahier Canadiens de Sociologie-*
Canada	*Canadian Review of Sociology and Anthropology—Revue Canadienne de Sociologie et d’Anthropologie*
Canada	*Canadian Review of Sociology—Revue Canadienne de Sociologie*
China	*Chinese Sociological Review*
China	*Chinese Sociology and Anthropology*
Croatia	*Društvena istraživanja*
Europe	*Archives Européennes de Sociologie*
France	*L’Homme & la société*
France	*Revue française de sociologie*
France	*Sociologie du Travail*
Germany	*Berliner Journal für Soziologie*
Germany	*Kölner Zeitschrift für Soziologie und Sozialpsychologie*
Germany	*Soziale Welt*
Germany	*Zeitschrift für Soziologie*
Ireland	*Social Studies Irish Journal of Sociology*
Italy	*Revista de Cercetare si Interventie Sociala*
Lithuania	*Filosofija. Sociologija*
Nordic Countries	*Acta Sociologica*
Poland	*Polish Sociological Bulletin*
Poland	*Polish Sociological Review*
Poland	*Studia Socjologiczne*
Russia	*Sotsiologicheskie issledovaniya*
Slovakia	*Sociología*
Slovakia	*Sociologický časopis/Czech Sociological Review*
Spanish speaking countries	*Convergencia. Revista de Ciencias Sociales*
Spanish speaking countries	*Revista Española de Investigaciones Sociológicas*
Spanish speaking countries	*Revista Internacional de Sociología*
Sweden	*Sociologisk Forskning*
