# Supplementary material for: The Prestige Elite in Sociology: Toward a Collective Biography of the Most Cited Scholars (1970-2010)
Source: Sociol Q. 2019 Apr 16;61(1):128–63. doi: 10.1080/00380253.2019.1581037 (PMC7077350; doi:10.1080/00380253.2019.1581037)
Supplement: Supplemental Material [file UTSQ_A_1581037_SM2619.docx]

**ONLINE SUPPLEMENT TO THE ARTICLE**

**The Prestige Elite in Sociology: Towards a Collective Biography of the Most Cited Scholars (1970-2010)**

Philipp Korom

*University of Graz*

**Codebook**

The article’s data were gathered in the framework of the project “Academic Super-Elites in Sociology and Economics” (P 29211) that was supported by the *Austrian Science Fund* (FWF). In the following, I describe the data structure as well as all variables contained in the dataset. When the data is used for theses or other scientific publications, the original data source should be cited as follows:

Korom, Philipp, Antonia Schirgi and Thomas Klebel. 2019. The Prestige Elite in Sociology (1970-2010) – A Citation Approach: Data file and code book. Retrieved from: [**please insert URL**]

The excel file contains eight worksheets, each referring to a different text source. These are the abbreviations used for naming each worksheet:

| **Worksheet** | **Source [source abbreviation]** |
| --- | --- |
| Sills_1970 | Sills, David L. ed. (1968) *International Encyclopedia of the Social Sciences.* New York: The Macmillan Company and the Free Press [Sills] |
| Wright_2010 | Wright, James D., ed. (2015) *International Encyclopedia of the Social & Behavioral Sciences.* Amsterdam: Elsevier [Wright] |
| Faris_1970 | Faris, Robert E., ed. (1964) *Handbook of Modern Sociology*. Chicago: Rand McNally [Faris] |
| Calhoun_2010 | Calhoun, Craig J., Chris Rojek and Bryan S. Turner, ed. (2005) *The Sage Handbook of Sociology*. London: Sage Publications [Calhoun] |
| JSTOR_1970 | All journal articles published in the *American Journal of Sociology* (AJS), the *American Sociological Review* (ASR), *Social Forces* (SF), the *British Journal of Sociology* (BJS) and the *European Journal of Sociology* (EJS) between 1970 and 1979. All articles were downloaded from JSTOR´s DFR site |

| **Worksheet** | **Source [source abbreviation]** |
| --- | --- |
| JSTOR_2010 | All journal articles published in the *American Journal of Sociology* (AJS), the *American Sociological Review* (ASR), *Social Forces* (SF), the *British Journal of Sociology* (BJS), and the *European Journal of Sociology* (EJS) between 2000 and 2009. All articles were downloaded from JSTOR’s DFR site |
| textbooks_1970 | Bierstedt, Robert (1974) *The Social Order: An Introduction to Sociology.* New York: McGraw-Hill [Bierstedt]  Broom, Leonard and Philip Selznick (1973) *Principles of Sociology. A Text with Adapted Readings.* New York: Harper & Row [Broom]  Horton, Paul B. and Chester L. Hunt (1964) *Sociology*. New York: McGraw-Hill [Horton]  Inkeles, Alex (1964) *What is Sociology? An Introduction to the Discipline and Profession*. Englewood Cliffs, New Jersey: Prentice-Hall [Inkeles]  Lenski, Gerhard (1970) *Human Societies: A Macrolevel Introduction to Society*. New York: McGraw-Hill [Lenski] |
| textbooks_2010 | Giddens, Anthony and Philip W. Sutton (2009) *Sociology*. Cambridge: Polity Press [Giddens]  Henslin, James M. (2015) *Essentials of Sociology: A Down-To-Earth Approach.* Boston: Pearson [Henslin]  Kendall, Diana E. (2011) *Sociology in Our Times*. Belmont, CA: Wadsworth/Cengage Learning [Kendall]  Macionis, John J. (2012) *Sociology*. Boston: Pearson [Macionis]  Schaefer, Richard T. (2013) *Sociology: A Brief Introduction*. Dubuque, Iowa: McGraw-Hill [Schaefer] |

The different worksheets contain the following variables:

**Encyclopedias**

| **Variable name** | **Meaning** |
| --- | --- |
| source | The text source |
| years | The time period covered |
| score_unweighted | Unweighted score for a reference to author; dummy variable; zero values indicate that references to an author are not made in the text source |
| second_name | Second name of the referenced author |
| first_name | First name of the referenced author |
| no_coauthors | Number of co-authors |
| vol | Volume of the encyclopedia |
| page | Page in the volume on which the reference can be found |
| enc_entry | Title of the encyclopedia entry |
| score_weighted | Score for a reference to author weighted by the number of all authors; numeric variable; zero values indicate that references to an author are not made in the text source |
| enc_entry_no | Continuous number of all encyclopedia entries |
| person_no | Continuous number of all authors considered |

**Handbooks**

| **Variable name** | **Meaning** |
| --- | --- |
| source | The text source |
| years | The time period covered |
| score_unweighted | Unweighted score for a reference to author; dummy variable; zero values indicate that references to an author are not made in the text source |
| second_name | Second name of the referenced author |
| first_name | First name of the referenced author |
| no_coauthors | Number of co-authors |
| page | Page in the handbook on which the reference can be found |
| chapter | Title of the handbook chapter |
| score_weighted | Score for a reference to author weighted by the number of all authors; numeric variable; zero values indicate that references to an author are not made in the text source |
| chapter_no | Continuous number of all handbook chapters |
| person_no | Continuous number of all authors considered |

**Journal Articles**

| **Variable name** | **Meaning** |
| --- | --- |
| source | The journal |
| years | The time period covered |
| score_unweighted | Unweighted score for a reference to author; dummy variable; zero values indicate that references to an author are not made in the text source |
| second_name | Second name of the referenced author |
| first_name | First name of the referenced author |
| no_coauthors | Number of co-authors |
| vol | Volume of the journal |
| page | Page in the volume on which the reference can be found |
| journal_article | Title of the journal article |

| **Variable name** | **Meaning** |
| --- | --- |
| score_weighted | Score for a reference to author weighted by the number of all authors; numeric variable; zero values indicate that references to an author are not made in the text source |
| journal_article_no | Continuous number of all journal titles |
| person_no | Continuous number of all authors considered |

**Textbooks**

| **Variable name** | **Meaning** |
| --- | --- |
| source | The textbook |
| years | The time period covered |
| score_unweighted | Unweighted score for a reference to author; dummy variable; zero values indicate that references to an author are not made in the text source |
| second_name | Second name of the referenced author |
| first_name | First name of the referenced author |
| no_coauthors | Number of co-authors |
| score_weighted | Score for a reference to author weighted by the number of all authors; numeric variable; zero values indicate that references to an author are not made in the text source |
| person_no | Continuous number of all authors considered |
